# Supplementary material for: Complexome profiling of the Chlamydomonas psb28 mutant reveals TEF5 as an early PSII assembly factor
Source: Plant Cell. 2025 Mar 17;37(6):koaf055. doi: 10.1093/plcell/koaf055 (PMC12164590; doi:10.1093/plcell/koaf055)
Supplement: koaf055_Supplementary_Data [file koaf055_supplementary_data.zip › psb28_interactive.zip!/plots/intensity/qualityPlots/peptides/NUO7_Cre09.g405850.t1.1.html]

### Cre09.g405850.t1.1

Trivial name: NUO7  
SeqCoverage [median]: 36.60  
Mass: 52.61 kDa  
  
 

| Sequence | MissedCleavage | Mass [Da] | wtIntensity | muIntensity | Score |
| --- | --- | --- | --- | --- | --- |
| ASGIDWDLR | 0 | 1031.50 | 1521.70 | 0.00 | 56.73 |
| ASGIDWDLRK | 1 | 1159.60 | 8391.64 | 12278.99 | 100.48 |
| AVEAPKGEFGVYLVSR | 1 | 1720.91 | 2052.82 | 11905.13 | 99.57 |
| EKLFEFYER | 1 | 1259.62 | 1744.03 | 1561.69 | 51.09 |
| GEFGVYLVSR | 0 | 1125.58 | 32595.28 | 37505.13 | 150.53 |
| LDEMEELLTGNR | 0 | 1418.67 | 18943.54 | 18694.37 | 269.01 |
| LFEFYER | 0 | 1002.48 | 24292.19 | 21357.21 | 108.24 |
| LVLEMQGEIIMR | 0 | 1430.76 | 15462.99 | 24896.94 | 190.26 |
| LYTEGFHVPAGETYR | 0 | 1738.83 | 33729.15 | 58584.39 | 114.64 |
| SPGYAHLQMTDVISR | 0 | 1673.82 | 33593.26 | 29328.27 | 194.77 |
| TQPYDAYGK | 0 | 1041.48 | 2576.60 | 0.00 | 64.27 |
| TYLQGLPYFDR | 0 | 1371.68 | 6013.29 | 0.00 | 96.89 |
| VGGVSQDLPIGLLR | 0 | 1422.82 | 23680.54 | 23553.45 | 246.26 |
| VLFSEITR | 0 | 963.54 | 25278.89 | 16113.52 | 109.86 |
| VNNFTLNFGPQHPAAHGVLR | 0 | 2188.13 | 23943.91 | 30372.92 | 111.06 |
|  |  |  | 253819.9 | 286152.0 |  |
